# More than an “inverted-U”? An exploratory study of the association between the catechol-o-methyltransferase gene polymorphism and executive functions in Parkinson’s disease

**DOI:** 10.1371/journal.pone.0214146

**Published:** 2019-03-21

**Authors:** Yi-Jia Fang, Chun-Hsiang Tan, Shao-Ching Tu, Chien-Yu Liu, Rwei-Ling Yu

**Affiliations:** 1 Institute of Behavioral Medicine, College of Medicine, National Cheng Kung University, Tainan, Taiwan; 2 Department of Neurology, Kaohsiung Medical University Hospital, College of Medicine, Kaohsiung Medical University, Kaohsiung, Taiwan; 3 Graduate Institute of Clinical Medicine, College of Medicine, Kaohsiung Medical University, Kaohsiung, Taiwan; 4 School of Medicine, College of Medicine, Kaohsiung Medical University, Kaohsiung, Taiwan; 5 Institute of Allied Health Sciences, College of Medicine, National Cheng Kung University, Tainan, Taiwan; 6 Department of Neurology, National Taiwan University Hospital, College of Medicine, National Taiwan University, Taipei, Taiwan; Universita degli Studi di Napoli Federico II, ITALY

## Abstract

Executive dysfunction is common in Parkinson’s disease (PD) patients. The catechol-O-methyltransferase (COMT) Val158Met polymorphism has been proposed to affect executive functions (EFs) in the prefrontal cortex. The present study attempted to explore the influence of the COMT polymorphism on EFs in patients with PD. Fifty-four PD patients were recruited and underwent neuropsychological assessments for three core EFs. The COMT polymorphism was genotyped using the TaqMan SNP Genotyping Assay. Participants were divided into three study groups: Val homozygotes, heterozygotes, and Met homozygotes. The three COMT genotype groups had significantly different performances in set-shifting [χ^2^ (2, 54) = 9.717, *p* = 0.008] and working memory tasks [χ^2^ (2, 54) = 7.806, *p* = 0.020]. Post-hoc analyses revealed that PD Val homozygotes performed significantly poorer in the set-shifting task than did either the PD Met homozygotes (*z* = -2.628, *p* = 0.009) or PD heterozygotes (*z* = -2.212, *p* = 0.027). Our explorative results suggest that the putative level of prefrontal dopamine influenced set-shifting through a “cane-shaped” dopamine level-response relationship. Our results have clinical implications, which may influence PD treatment with dopamine in the future because the optimal dopamine level to maximize EFs may vary based on the clinical course and COMT polymorphism status. Further study recruiting a larger number of participants is needed to confirm our preliminary findings.

## Introduction

Parkinson's disease (PD) is one of the most common neurodegenerative disorders. The prevalence of PD in Taiwan in 2011 was 147.7 per 100,000 person-years. The prevalence and incidence of PD increase with age [1]. PD is characterized by the death of dopaminergic neurons in the substantia nigra pars compacta, predominantly in its ventrolateral tier [2]. The loss of dopaminergic neurons in this area may lead to the development of motor symptoms [2], including bradykinesia, rigidity, resting tremor, and postural instability [3].

In addition to motor symptoms, impaired cognitive functions are also recognized as one of the features of PD. In fact, among cognitive impairments, executive dysfunctions are very common in PD [4]. A previous study reported that up to 59.1% of PD patients with mild cognitive impairments exhibit executive dysfunction [4]. Executive functions can be subdivided into core and higher-order executive functions [5]. The former includes cognitive flexibility (also called set-shifting), working memory, and inhibition, while the latter includes reasoning, problem-solving, and planning.

To counter the effect of dopaminergic neural deaths, pharmacological agents such as levodopa, dopamine agonists, and monoamine oxidase type B inhibitors (MAO-B inhibitors) have been used to increase the intracerebral dopamine level or to stimulate the dopamine receptors in PD patients. Dopamine plays a crucial role in modulating executive functions [6]. Studies have reported that dopaminergic drugs could either enhance or impair working memory among different individuals performing the same task. In other words, too little or too much dopamine has deleterious effects on performance [7]. Therefore, the hypothesis of an inverted-U shaped relationship between dopamine levels and working memory has been widely accepted [7].

Results from animal studies have indicated that the “inverted-U” shaped dopamine level-response relationship does not fit all executive functions [8]. Floresco [8] demonstrated an exception to the inverted-U rule: set-shifting, which involves activities of dopamine D1 and D2 receptors. In their study, while blockade of D1 and D2 receptors impaired set-shifting, pharmacological stimulation (SKF81297 or Quinpirole) of these receptors did not affect performances in set-shifting. Therefore, in this study, Floresco discovered an executive function for which the inverted-U rule did not apply. However, the results and inferences were drawn from studies in rodents, which may raise some uncertainties in their implications in humans.

The catechol-O-methyltransferase (COMT) regulates dopamine levels in cortical areas [9]. The peptide bond sequence of the COMT gene may change at the position of codon 158, turning valine (Valine, Val) into methionine (Methionine, Met). Val homozygotes (Val/Val) code for COMT with 40% higher enzymatic activity in dopamine metabolism than do Met homozygotes (Met/Met), likely resulting in a reduction in brain dopamine [10]. Thus, this COMT Val158Met gene polymorphism has been proposed to affect executive functions [11].

Many studies have found that the COMT Val158Met gene polymorphism affects the performance of executive functions in PD patients. In terms of planning ability, previous studies [11, 12] hypothesized that based on the inverted-U rule, PD Met homozygotes, which code for COMT with poor dopamine metabolism, were postulated to have poorer planning performances compared to those of PD Val homozygotes, due to the hyperdopaminergic state in the prefrontal cortex (PFC) in early PD. As the disease progresses, the prefrontal dopamine level decreases. The performance of PD Met homozygotes may improve while that of PD Val homozygotes may deteriorate [12, 13]. However, studies using the Tower of London test did not have consistent results [12–14]. This inconsistency may be due to demographic variables, different cut-off points of disease duration, or different forms of the test utilized by the studies.

Two studies on cognitive control [15, 16] have shown that patients with early-stage PD who were Met homozygotes appeared to have poorer performances in set-like behavior than did patients with early-stage PD who were Val homozygotes. Fallon et al. [17] demonstrated that among three COMT genotypes, patients with heterozygotes had the best performance, in both spatial working memory task and set-shifting task. Although the PD Met homozygotes seemed to have made more errors during the spatial working memory task than in the set-shifting task, no significant difference was found in relative task performances between the PD heterozygotes and PD Val homozygotes. This result was not affected by disease duration, medications, or age. Fallon et al. [17] then proposed that prefrontal dopamine levels influenced spatial working memory in an “inverted-U”-shaped fashion, while a linear pattern was observed for set-shifting. Furthermore, they presumed that prefrontal dopamine might not influence cognition uniformly.

To date, no study has demonstrated a significant difference in inhibitory function among PD patients with different COMT genotypes. Leroi et al. [18] used a computerized test to examine inhibition abilities in PD patients, and the results showed that there was no significant difference in the response time or error rate among patients with different COMT genotypes.

It seemed that previous studies have not yet concluded and that most of the research only focused on one subcomponent of executive functions. Therefore, the present study aimed to explore the effect of COMT polymorphism on different executive functions in PD patients. The present study selected neuropsychological tests sensitive to three core executive functions and hypothesized that PD patients with different COMT gene polymorphisms had different performances in executive function. First, we hypothesized that the relationship between set-shifting and the dopamine level followed a cane-shaped curve, in which poor set-shifting performances were related to lower dopamine levels. Secondly, the relationship between working memory scores and the dopamine level in the prefrontal cortex followed the inverted-U shape, in which poor working memory was associated with both higher and lower dopamine levels. Finally, the level of frontal dopamine did not affect the inhibition performance.

## Methods

### Participants

Fifty-four PD patients were included in this study. Only patients with idiopathic PD (according to the UK PD Society Brain Bank criteria) and normal visual acuity were included in the study. Young-onset PD patients with motor symptom onset before 50 years of age, patients with atypical features of parkinsonism, a history of brain surgery, severe systemic diseases, and psychiatric diseases (e.g., depression, schizophrenia, etc.), and patients who were illiterates were excluded from the study. All participants provided written informed consent before enrolment by the ethical standards established in the 1964 Declaration of Helsinki. All study procedures were approved by the ethical research committee of Kaohsiung Medical University Memorial Hospital (KMUHIRB-G(I)-20160001).

### Clinical characteristics

The age, age at onset, years of education, disease duration, Modified Hoehn and Yahr Staging Scale score, and levodopa equivalent dose [19] of patients in each group were collected.

### Neuropsychological assessment and genotyping

#### Neuropsychological assessment

This study utilized the Mini-Mental State Examination (MMSE) [20] to assess global cognitive function. A series of tests were also conducted to assess executive function. Modified Wisconsin Card Sorting Test (M-WCST) [21] and Semantic Association of the Verbal Fluency Test [22] were used to assess set-shifting. The Digit Span Subtest of WAIS-Chinese Revision [23] and the Working Memory Index of WAIS-Chinese Revision [23] were used to assess working memory. The Stroop Word-Colour Test [24] was used to assess inhibitory functions.

#### Genotyping

Genetic testing was performed after a neuropsychological evaluation. DNA was extracted from peripheral blood leukocytes using the Genomic DNA Extraction Kit (Geneaid, New Taipei City, Taiwan). COMT Val158Met (SNP rs4680) was genotyped using a TaqMan SNP Genotyping Assay.

### Statistical analysis

Experimental design: tested subjects were assigned to the corresponding study groups based on their genotypes (Val homozygotes, Val/Met heterozygotes, and Met homozygotes). The results from each study group were compared using the Kruskal-Wallis test for nonparametric variables. The Dunn’s multiple comparison tests were performed if a significant difference in results was found among the genotypes. Statistical significance was defined as *p* < 0.05.

## Results

The demographic data are summarized in Table 1. Demographic variables, including patient age, age at onset, levodopa equivalent dose, years of education, and disease duration, revealed no significant difference among the three study groups (*p* > 0.05).

**Table 1:** Demographic characteristics and mental state of study groups.

	Val/Val (n = 33)		Val/Met (n = 15)		Met/Met (n = 6)		Stat. Value	*p-*value
	*Mean*	*SD*	*Mean*	*SD*	*Mean*	*SD*		
**Gender, F/M**	10/23	-	7/8	-	2/4	-	-	-
**Age, y**	63.70	4.96	63.93	8.80	59.5	4.81	2.88	0.24
**Education, y**	11.70	4.48	12.27	4.32	13.50	3.99	0.78	0.68
**Onset**	59.39	5.32	59.93	9.44	54.50	2.07	3.84	0.15
**Disease duration, y**	4.39	2.79	4.13	2.92	5.00	3.16	0.48	0.79
**Hoehn and Yahr Stages**	1.66	0.60	1.73	0.88	1.67	0.52	0.03	0.99
**Levodopa equivalent dose**	567.00	256.55	584.96	263.80	790.25	385.51	1.71	0.43
**MMSE**	28.09	2.08	28.00	2.20	28.17	2.14	0.02	0.99

Abbreviations: SD, standard deviation; Stat. Value, statistical value; MMSE, Mini-Mental State Examination

The results of the executive function tests are shown in Table 2. There was a significant effect of COMT genotype on non-perseverative errors in the M-WCST [χ^2^ (2, *N* = 54) = 9.717, *p* = 0.008] and on the score on the Digit Span Subtest-Backward of Wechsler Adult Intelligence Scale (WAIS)-Chinese Revision [χ^2^ (2, *N* = 54) = 7.806, *p* = 0.020]. Post-hoc analyses indicated that in the M-WCST, the PD Val homozygotes made more non-perseverative errors (M-WCST-NP) than both the PD Met homozygotes (*z* = -2.628, *p* = 0.009) and PD heterozygotes (*z* = -2.212, *p* = 0.027). The post-hoc analysis also showed that in the Digit Span Subtest-Backward, the performance of PD Val homozygotes was worse than that of the PD Met homozygotes (*z* = 2.540, *p* = 0.011).

**Table 2:** Executive functions in the study groups.

	Val/Val		Val/Met		Met/Met		Stat. Value	*p*-value
	*Mean*	*SD*	*Mean*	*SD*	*Mean*	*SD*		
**M-WCST**	-	-	-	-	-	-	-	-
**M-WCST-C**	4.12	2.09	4.60	2.10	6.17	0.98	5.08	0.079
**M-WCST-P**	4.36	3.89	7.47	12.19	1.17	1.47	4.36	0.113
**M-WCST-NP**	9.73	5.00	6.13	3.42	4.67	2.07	9.72	**0.008**
**Semantic verbal fluency test**	34.00	8.803	34.07	7.54	36.50	9.31	0.18	0.912
**Digit Span**	10.91	2.24	11.60	2.92	11.67	2.58	0.78	0.679
**Forward**	7.82	1.16	7.80	1.32	7.67	0.82	0.49	0.783
**Backward**	4.21	1.14	4.93	1.58	5.83	1.33	7.81	**0.020**
**Working Memory Index**	102.30	13.42	104.67	15.34	110.17	7.731	1.64	0.440
**Stroop test**	-	-	-	-	-	-	-	-
**Interference**	-3.09	7.07	0.58	7.70	-1.55	4.97	3.28	0.194

Abbreviations: SD, standard deviation; Stat. Value, statistical value; M-WCST-C, M-WCST-P, and M-WCST-NP indicate the achieved categories, perseverative errors, non-perseverative errors in the Modified Card Sorting Test, respectively.

**Fig 1:**
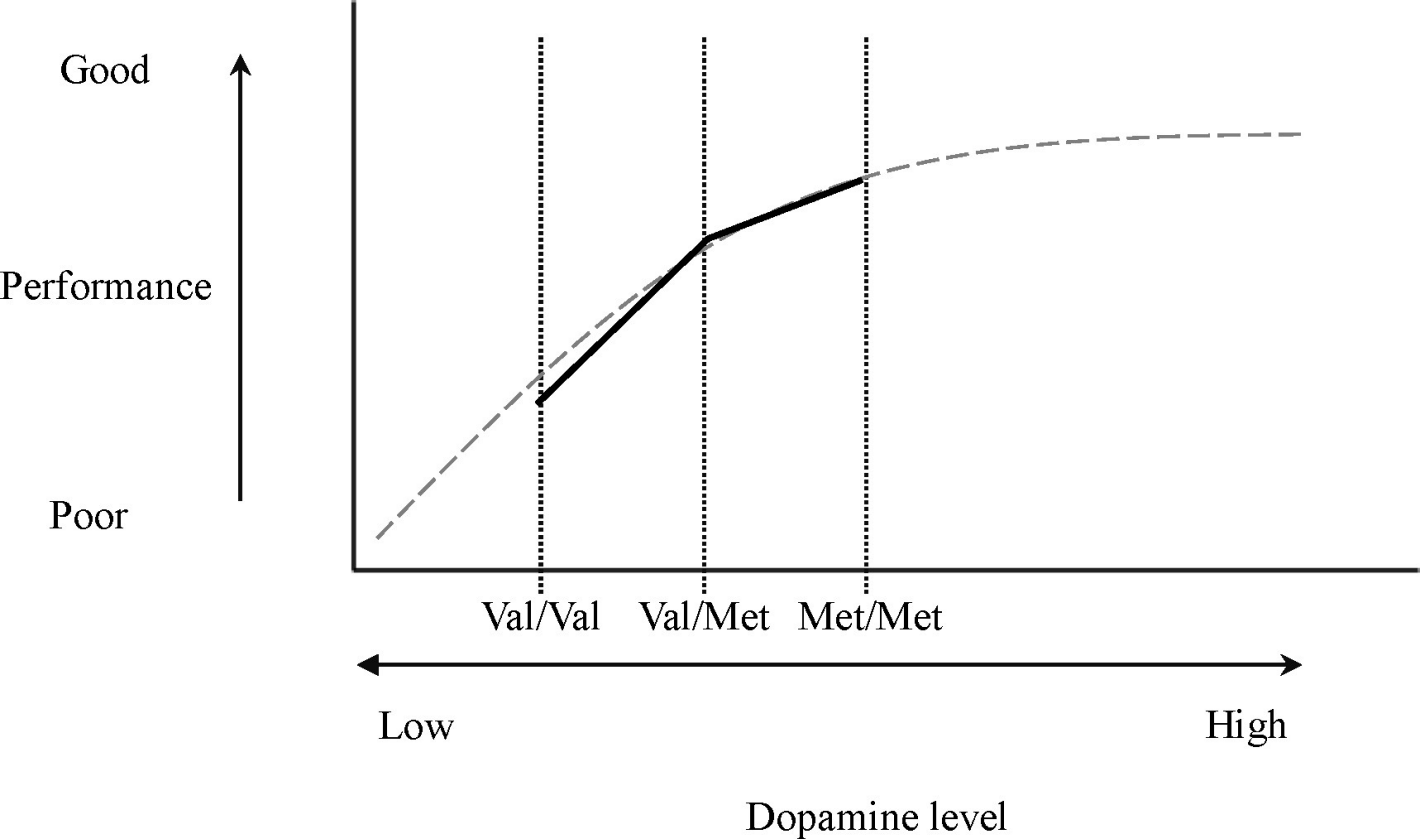
The speculated “cane-shaped” curve of association between set-shifting and dopamine level in PD. The gray dotted line represented the association between set-shifting and dopamine level, which was based on Floresco [8]. The vertical black dotted lines represented the dopamine levels of COMT Val158Met polymorphism. The solid black line indicated the set-shifting performance in the study groups.

Based on our data, we draw a figure (Fig 1) to show the relationship between the set-shifting and dopamine level in patients with PD.

**Fig 2:**
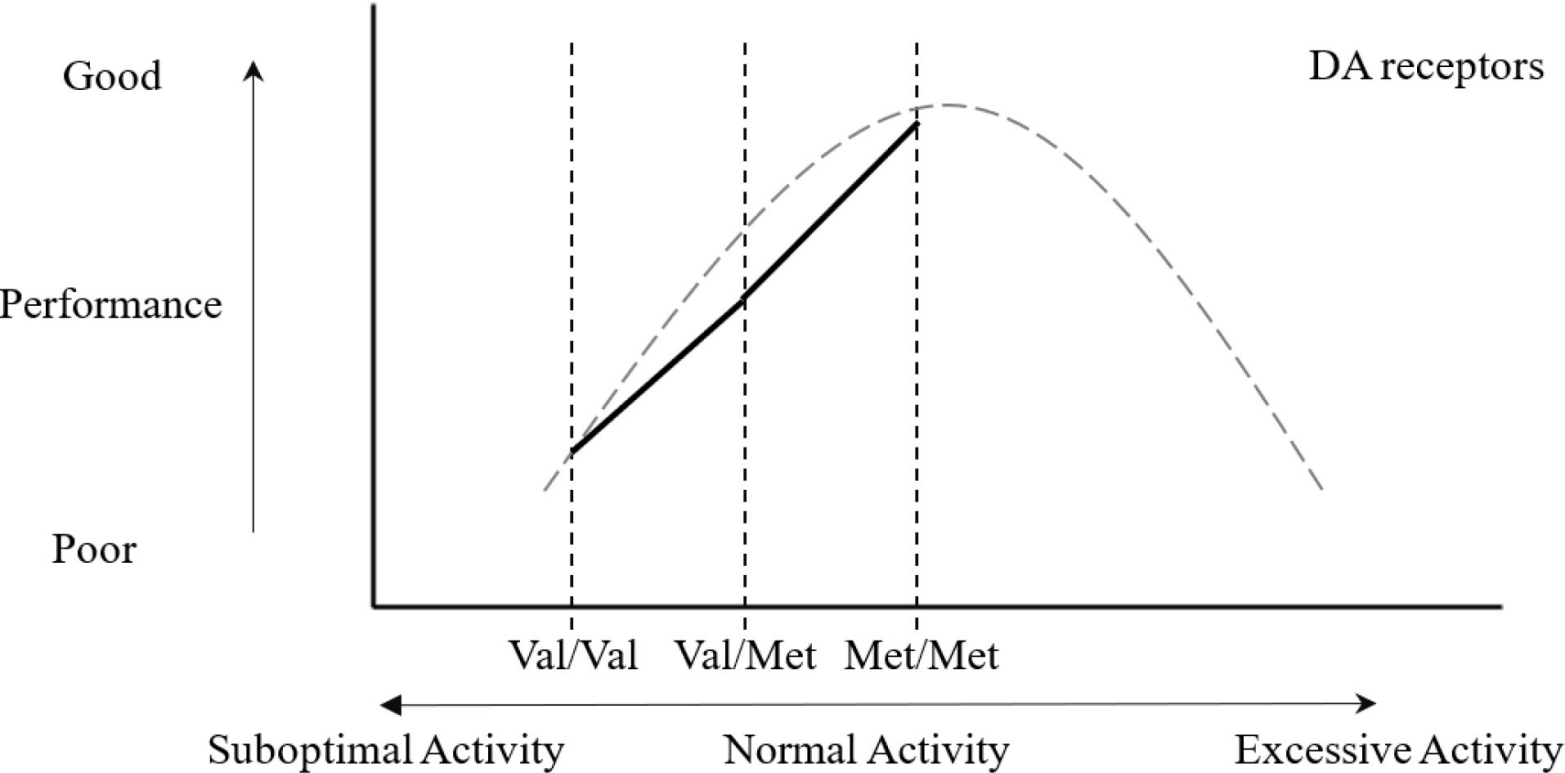
The “inverted-U” curve of association between working memory and dopamine level in PD. The gray dotted line represented the association between working memory and dopamine level, which was based on Cools and D'Esposito [7]. The vertical black dotted lines represented the dopamine levels of COMT Val158Met polymorphism. The solid black line indicated the working memory performance in the study groups.

Also, Fig 2 were showed to represent the association between the working memory and dopamine level of patients with PD.

## Discussion

This study aimed to explore the effect of the COMT Val158Met polymorphism on various executive functions in PD patients. The current study presents a group of PD patients, presumed to be in the early stages of the disease. The clinical course of PD may be characterized by changes in dopamine level, which may be modulated by a combination of two variables: 1) the extent of damage to the dopaminergic system and 2) the activity of COMT enzyme which is coded by a specific genotype in an individual. In the early stages of the disease, minor dopaminergic system damage may be supplemented by higher dopamine levels due to the lower activity level of the COMT enzyme coded by Met homozygotes. This is observed from the high performance of patients in the Digit Span Subtest-Backward task and less non-preservative errors made in the M-WCST. This implies that due to the relatively insufficient dopamine level attributable to the higher activity level of COMT, patients carrying Val homozygotes, performed poorly in the set-shifting and working memory tasks.

The explorative results indicate that in the M-WCST, PD Val homozygotes made more non-preservative errors than both the PD Met homozygotes and PD heterozygotes, and that no significant difference was found between PD Met homozygotes and PD heterozygotes. The result of the present study was partially compatible with the results of a previous study [17], which also indicated that PD Val homozygotes performed worst in the set-shifting task. Animal studies [8] have shown that during the higher activation state of dopamine D1 and D2 receptors, the performance of rats in the set-shifting task did not decline. However, performance in the set-shifting tasked declined when prefrontal dopamine receptors were in the low activity state. The researcher plotted a “cane-shaped” curve to explain this phenomenon. Our study also suggested that both PD Met homozygotes and PD heterozygotes performed better in the set-shifting task compared to PD Val homozygotes, which supports the cane-shaped relationship by associating lower dopamine levels with impaired ability in the set-shifting task. A higher dopamine level, on the other hand, did not impair performance in the set-shifting task (Fig 1).

Results from previous studies [15, 16] could not be reproduced in our study. Williams-Gray et al. [16] suggested that impairment in maintaining attention was observed early in PD Met homozygotes, a conclusion that was inconsistent with our findings. The tasks that were used by Williams-Gray et al. included the intra-dimensional task (ID task) and the extra-dimensional task (ED task). The former measured set formation, while the latter measured set-shifting. The results from the ED task seemed to have indicated that the PD Met homozygotes performed better than the PD Val homozygotes. However, the study lacked detailed statistical data in this part of the study. Fallon et al. [15] also used the ID/ED tasks to investigate set-like behaviors in various COMT polymorphism PD patients, but the results were not consistent with those of the current study. In their study, the difference between the number of ED and ID errors was considered to indicate the level of set-like behavior. A larger discrepancy suggested better set-like behavior. Therefore, the authors suggested that the PD Val homozygotes had a better level of set-like behavior than did the PD Met homozygotes. However, this conclusion was based on a calculation, which might have missed the original meaning of ED and ID error values. It would have been better if the authors also discussed the raw data for these two values. For the reasons listed, confirming the validity of the conclusion that set-shifting in PD Val homozygotes is superior to that in PD Met homozygotes, still requires further investigation.

Regarding working memory, the results from the Digit Span subtest indicated that PD Met homozygotes had significantly superior working memory performance compared to PD Val homozygotes. Our results fit the left side of the “inverted-U” shaped curve underlying working memory and the dopamine level (Fig 2), a result similar to that of a previous study [17, 25]. The level of dopamine in PD Val homozygotes was relatively lower than that in both PD heterozygotes and Met homozygotes. Therefore, relatively impaired performance in working memory was expected. However, no significant difference was found among the three groups in the Working Memory Index of WAIS-Chinese Revision, presumably because the Working Memory Index included an arithmetic subtest, which required reasoning ability and calculating skills that might have confounded the result.

In the current study, performance in the Stroop Word-Colour Test were not significantly different among PD patients with various COMT gene polymorphisms, which might imply that other neurotransmitters may be more dominant in modulating inhibitory functions, an inference which is consistent with findings from previous studies [14, 18]. One review article [26] suggested that inhibition ability in rats was not affected by dopamine D1/D2 antagonists, and that norepinephrine transmission in the PFC is related to inhibitory functions. Ye et al. [27] suggested that selective serotonin reuptake inhibitors could improve behavioral impulsivity, action restraint, and behavior cancellation in PD. Therefore, we speculated that while dopamine did not seem to dominate inhibitory functions, other neurotransmitters such as norepinephrine and serotonin might play a more critical role in modulating inhibitory abilities.

At present, treatments that help improve cognitive dysfunction in PD patients include medical treatments. Some studies explored the efficacy of AD medications (such as cholinesterase inhibitor or MAO-B inhibitor) in treating mild cognitive impairment in PD patients. After evaluating patients for six months, several neuropsychological tests did not show significant changes in the cognitive functions of patients [28, 29]. Other studies have investigated the effect of dopamine on cognitive function in PD. For example, Poletti and Bonuccelli [30] reviewed articles to explore the effects of levodopa and dopamine agonists on cognitive function in PD patients, but their conclusion remained inconsistent. It may be because the effect of the COMT Val158Met polymorphism was not considered in previous studies. We suggest that future treatments should involve personalized drug dosages based on the COMT activity and the resultant dopamine level in the brains of patients. Decoding genetic codes, such as that for the Val158Met polymorphism, may help to maximize the therapeutic effects of medication. This will enable patients to receive personalized treatments to preserve motor and executive functions.

The first limitation of this study were the small sample size, no healthy comparison group, and the large discrepancy between the number of Val homozygotes patients and Met homozygotes patients. According to the Ensembl genome database which recruits the data from all over the world, the frequency of the Met allele is only 0.280 in the East Asian population [31] and 0.225 to 0.259 in Taiwanese population [32], which is lower than that among Europeans (0.50) and Americans (0.378) [31]. The frequency of Met homozygotes in East Asia is only 0.083 [31], and we further compared the frequency of occurrence of polymorphisms in other studies concerning the same population and found similar frequency [32–34]. Studies in East Asia could only access less than ten participants of Met homozygotes even though they have more than one hundred participants. Also, the demographic data of the present study revealed there is no significant difference among three groups, especially the disease duration of PD, Levodopa equivalent dose, and MMSE, which made the study more convincing. Admittedly, the current study is an exploratory study and our result are based on a small sample size. Future research recruiting a larger number of participants is required to validate, refute, or modify our preliminary results. Second, the present study failed to explore higher-order executive functions, such as reasoning, problem-solving, and planning. Executive functions are complicated and comprise a core and higher-order executive functions. We clarified the relationship between core executive functions and COMT genotypes, thereby helping to explore the relationship between higher-order executive functions and COMT genotypes. Future studies can use a larger sample size and examine higher-order executive functions in order to overcome these challenges.

In conclusion, our results indicate that executive functions such as set-shifting and working memory in PD are influenced by COMT gene polymorphism, while inhibition is not affected by the COMT gene polymorphism. These results add a piece to the puzzle by uncovering the effect of prefrontal dopamine levels on working memory and set-shifting in the early stages of PD. Future studies recruiting more participants, longer patient follow-ups, or comparing PD patients in different clinical stages may be needed to confirm our preliminary findings that the existence of an inverted-U shaped relationship between prefrontal dopamine levels and working memory, or a cane-shaped relationship between prefrontal dopamine levels and set-shifting.

## Supporting information

S1 File Plosonedata-20190305. Minimal dataset.
(XLSX)
